# Evaluation of processing of canola protein isolate on postprandial plasma amino acid profiles in healthy, young females

**DOI:** 10.1007/s00726-025-03482-1

**Published:** 2025-10-17

**Authors:** Noortje Boot, Wesley J. H. Hermans, Ines Warnke, Alex Overman, Janneau M. X. van Kranenburg, Joan M. Senden, Lex B. Verdijk, Luc J. C. van Loon

**Affiliations:** 1 Department of Human Biology, NUTRIM Institute of Nutrition and Translational Research in Metabolism, Department of Human Biology, Maastricht University Medical Centre+, PO box 616, Maastricht, 6200 MD The Netherlands; 2 Human Nutrition and Care (HNC) Innovation, R&D and Regulatory, dsm-firmenich, Kaiseraugst, AG Switzerland

**Keywords:** Plant-derived protein, Bioavailability, Protein processing

## Abstract

Canola protein is a rapeseed-derived protein that contains all essential amino acids in proportions that meet the WHO amino acid scoring requirements, making it an interesting protein for human food applications. It is currently unknown whether canola protein processing modulates postprandial plasma amino acid bioavailability in vivo in humans. This study compared postprandial plasma amino acid profiles following the ingestion of unprocessed (native) canola, processed canola, and whey protein isolate in healthy, young, females. In a randomized, clinical, cross-over design, 15 healthy young females (25 ± 3 y) participated in four test days on which they consumed 20 g protein as either native canola, enzyme processed or heat processed canola protein, or 20 g whey protein. Blood samples were collected for 5 h following protein ingestion to assess plasma amino acid concentrations. Ingestion of native canola protein resulted in lower increases in plasma total amino acid (TAA) concentrations compared to whey protein (3191 ± 794 vs. 4429 ± 84 µmol∙L^− 1^, *P* < 0.001). Canola protein processing resulted in greater peak plasma total amino acids concentrations, reaching statistical significance for enzyme (3599 ± 687 µmol∙L^− 1^, *P* = 0.045) but not heat (3565 ± 722 µmol∙L^− 1^, *P* = 0.166) treated compared to native canola protein. Plasma total amino acid availability, expressed as incremental area under the curve over a 5 h postprandial period, did not differ between treatments and averaged 163 ± 81, 171 ± 76, 194 ± 82, and 207 ± 85 mmol∙300 min∙L^− 1^ following ingestion of native, enzyme- and heat processed canola, and whey protein, respectively (*P > 0.05*). Ingestion of whey protein allows for a more rapid postprandial rise in circulating essential and non-essential amino acids and greater postprandial plasma total amino acid availability when compared to the ingestion of native canola protein. Ingestion of enzyme- or heat processed canola protein accelerates the postprandial rise in circulating amino acids but does not further augment overall plasma amino acid availability throughout a 5 h postprandial period when compared to the ingestion of native canola protein.

**Supplementary Information:**

The online version contains supplementary material available at 10.1007/s00726-025-03482-1.

## Background

Ingestion of dietary protein stimulates muscle protein synthesis (Boirie et al. 1997a, b; Groen et al. 2015; Trommelen et al. 2023; Moore et al. 2015). The anabolic response to protein ingestion is regulated at various levels starting from protein digestion, the absorption of free amino acids in the gastrointestinal tract, their (partial) release into the systemic circulation, and their uptake and subsequent incorporation into muscle protein (Boirie et al. 1997a, b; Groen et al. 2015; West et al. 2011; Gorissen et al. 2016; Volpi et al. 1999). Both the amount and type of protein that is ingested determine the postprandial rise in circulating amino acid concentrations (i.e. bioavailability) and, as such, modulate the anabolic response to protein ingestion (Gaudichon and Calvez 2021; Gorissen et al. 2020). Therefore, the postprandial plasma amino acid profile is often used as a proxy for the anabolic properties of a protein source.

With the transition towards more sustainable diets, there is an increased interest in the consumption of more plant-based products due to their lower environmental impact and lower risk of developing chronic metabolic diseases (Rocha et al. 2019; Musicus et al. 2022; Lamberg-Allardt et al. 2023). However, plant-derived proteins are considered to have lesser anabolic properties than animal-derived proteins such as whey or milk (Tang et al. 2009; Yang et al. 2012; Wilkinson et al. 2007). The lesser anabolic properties of plant-derived proteins have been attributed to lower digestibility, lower essential amino acid content, and/or deficiencies in one or more specific amino acids such as leucine, lysine, or methionine (Gorissen et al. 2018; Vliet et al. 2015; Pinckaers et al. 2021). Despite their lesser anabolic properties, plant-derived protein isolates and concentrates are increasingly being used in protein supplements and food products, such as meat and dairy analogues (Kotecka-Majchrzak et al. 2020; Jia et al. 2021). Due to the increased interest in plant-derived proteins, a number of recent studies have compared the postprandial plasma amino acid profiles following ingestion of various plant- versus animal-derived proteins (Gorissen et al. 2016; Tang et al. 2009; Yang et al. 2012; Pinckaers et al. 2022, 2023, 2024a, b). Collectively, these data show an attenuated amino acid response following the ingestion of soy, wheat, potato, corn, and pea protein when compared to the ingestion of equivalent doses of dairy or milk protein. The attenuated amino acid response following ingestion of plant- versus animal-derived proteins can be attributed to differences in protein structure and function that impact protein digestion and amino acid absorption (Groen et al. 2015; Trommelen et al. 2021; Kashyap et al. 2018, 2019).

The production of protein isolates requires several industrial processing techniques (i.e. physical and chemical) that may change protein structure and function. For example, the heating of milk protein beyond standard pasteurization conditions (> 85 °C for extended periods) induces protein glycation due to the Maillard reaction (Mauron 1981; Schmitz-Schug et al. 2013; Guyomarc’h et al. 2000). High levels of protein glycation result in the inability to absorb lysine (Nyakayiru et al. 2020), resulting in severely compromised plasma lysine availability (Lieshout et al. 2025). Similar to animal-derived proteins, plant-derived proteins may be susceptible to Maillard reactions during processing, and often contain anti-nutritional factors that may negatively affect protein bioavailability (Herreman et al. 2020; Moughan 2021). In contrast, other techniques may positively impact bioavailability. For example, boiling eggs denatures the available protein, resulting in more rapid and greater postprandial amino acid availability when compared to the ingestion of raw eggs (Evenepoel et al. 1998; Fuchs et al. 2022). In line, partial hydrolysis of micellar casein through enzyme treatment has been shown to accelerate protein digestion and augment the release of protein-derived amino acids in the circulation in vivo in older men (Koopman et al. 2009). Clearly, more work is required to establish the positive or negative impact of (industrial) processing of both animal and plant-derived proteins on postprandial bioavailability as well as bio-functionality.

Rapeseed is the world’s second most produced oilseed after soybean (Campbell et al. 2016; Sarwar et al. 2003). Cultivation of the crop is primarily done to produce canola oil. Canola press-cake, which is a by-product from the extraction of rapeseed oil, contains ~ 35–40% protein on a dry weight basis (Campbell et al. 2016; Wickramasuriya et al. 2015). Besides its high protein content, canola is a high-quality plant-based protein source due to its amino acid composition which meets human requirements (Gorissen et al. 2018). Recently it has been shown that ingestion of heat processed canola protein results in greater protein digestibility in pigs, when compared to the ingestion of native canola protein (Bailey et al. 2023). So far, no data are available on the postprandial plasma amino acid response following native canola protein ingestion in vivo in humans. Furthermore, it remains to be assessed whether processing of native canola protein, via heat or enzyme treatment, modifies the postprandial plasma amino acid response. We hypothesized that heat and enzyme processing facilitate and accelerate protein digestion and amino acid absorption, resulting in a more rapid postprandial rise in circulating amino acid concentrations. To test our hypothesis, fifteen healthy, young females were recruited to participate in a clinical cross-over study in which we assessed the plasma amino acid responses following the ingestion of 20 g native canola protein isolate, 20 g heat-treated canola protein isolate, 20 g enzyme-treated canola protein isolate, and 20 g of a reference whey protein isolate throughout a 5 h postprandial period.

## Methods

### Participants

Fifteen healthy, young female subjects volunteered to participate in this randomized double-blind, clinical cross-over study (Table 1 for participants’ characteristics). All procedures involving human participants were in accordance with the ethical standards of the institutional and/or national research committee and with the 1964 Helsinki Declaration and its later amendments or comparable ethical standards. The study was approved by the Medical Ethical Committee of the Maastricht University Medical Centre+ (azM/UM), Maastricht, The Netherlands (Ethics approval number: METC23-025), and was registered at www.clinicaltrials.gov (NCT06058403). All experimental procedures were conducted between October 2023 and May 2024 at Maastricht University, The Netherlands. Participants were informed about the experimental procedures and possible risks of participation prior to signing informed consent. All participants provided written informed consent to participate in the study. The study was independently monitored by Clinical Trial Centre Maastricht.

**Table 1:** Participant’s characteristics

Parameter	Total (*n = 15*)		
Age (y)	25 ± 3		
Body mass (kg)	63.1 ± 6.7		
Height (m)	1.68 ± 0.1		
BMI (kg/m^2^)	22.3 ± 1.6		
Body fat (%)	27.8 ± 4.9		
Lean body mass (kg)	43.5 ± 4.6		
Appendicular lean mass (kg)	19.4 ± 2.3		
Systolic blood pressure (mmHg)	106 ± 9		
Diastolic blood pressure (mmHg)	66 ± 8		
Resting heart rate (bpm)	66 ± 12		

### Preliminary testing

Participants aged 18–35 y, with a BMI between 18 and 30 kg/m^2^ underwent an initial screening to assess eligibility, whereby body height (m), mass (kg) and blood pressure (mmHg) were determined. Participants were deemed healthy based on their response on a routine medical questionnaire. Potential subjects were included if they were non-smoking, recreationally active (exercise ≤ 3 times per week), and had no history of intolerance for dairy. Participants were excluded from participation if suffering from hypertension (> 140/90 mmHg), or gastrointestinal disorders, were smoking, participating in a progressive resistance-type exercise training program, using third generation oral contraceptives, or indicated intolerance to the investigational food products. Body composition was assessed by dual energy X-ray absorptiometry (DEXA). The screening session and the first trial day were separated by at least 3 d.

### Study design

In this randomized, cross-over design, participants (*n* = 15) ingested either 20 g canola protein isolate in its unprocessed (native) or processed (heat- or enzyme treated) form, or 20 g whey protein isolate. Subjects performed four test days, separated by at least three days. Arterialized blood samples were collected frequently to assess post-absorptive and postprandial plasma amino acid concentrations. Interventional drink allocation and all analyses were performed in double blinded manner.

### Standardization of diet and physical activity

Three days prior to the first experimental trial day, participants refrained from any sort of heavy physical activity and alcohol consumption. In the two days prior to the first trial day, participants recorded their dietary intake and physical activity. Prior to subsequent trial days, participants received copies of these records and adhered to their own dietary intake and physical activity level. On the evening prior to each of the 4 test days, all participants consumed the same standardized dinner providing 2.5 MJ, with 50 Energy (En) % carbohydrate, 29En% fat, and 16En% protein.

### Experimental procedures

The experimental trial day was scheduled in the first 10 d of the menstrual cycle for participants not taking hormonal contraceptives, which was assessed by self-report to control for hormonal fluctuations. At 07:45 AM, participants reported to the laboratory in an overnight fasted state (~ 10 h). A catheter was inserted into a dorsal hand vein for arterialized blood sampling. To obtain the arterialized blood, the hand was placed into a hotbox (60 ℃) for 10 min prior to every blood sample collection.

After taking a baseline blood sample (t = 0 min), 20 g protein (based on the sum of total amino acids, Table 2) was dissolved in 300 mL water. All test drinks were flavored with 3 mL vanilla flavor (Dr. Oetker, Amersfoort, The Netherlands) and provided in a non-transparent shaker and consumed within 5 min. Immediately after finishing the drink, a 5-hour postprandial period was initiated during which arterialized blood samples were collected at t = 15, 30, 45, 60, 90, 120, 180, 240, and 300 min. Blood samples were collected into EDTA-containing tubes and centrifuged at 1000*g* for 10 min at 4 ℃. Aliquots of plasma were frozen in liquid nitrogen and stored at -80 ℃ until later processing. After completion of the experimental protocol, the cannula was removed, and participants received a small meal before leaving the laboratory.

**Table 2:** Amino acid composition of protein isolates

	Whey	Native Canola	Canola heat	Canola enzyme
Alanine	0.9	0.8	0.8	0.8
Arginine	0.4	1.4	1.3	0.8
Aspartic acid	2.2	1.2	1.1	1.2
Cystine	0.4	0.7	0.7	0.3
Glutamic acid	3.6	4.9	5.0	5.0
Glycine	0.3	0.9	0.9	0.9
Histidine	0.3	0.7	0.7	0.7
Hydroxyproline	0.0	0.0	0.1	0.1
Isoleucine	1.3	0.7	0.7	0.7
Leucine	2.1	1.4	1.4	1.5
Lysine	1.9	1.3	1.3	1.3
Methionine	0.4	0.4	0.5	0.2
Phenylalanine	0.6	0.8	0.8	0.8
Proline	1.2	1.6	1.5	1.7
Serine	0.9	0.8	0.8	0.8
Threonine	1.4	0.7	0.7	0.7
Tyrosine	0.6	0.4	0.4	0.4
Valine	1.1	0.9	0.9	1.0
Tryptophan	0.4	0.3	0.3	0.3
Citrulline-Ornithine	0.0	0.0	0.0	0.6
Total NEAA	10.5	12.5	12.4	12.5
Total EAA	9.6	7.3	7.3	7.2
Total AA	20.1	19.8	19.8	19.8

Values for amino acid contents are in grams per 20 g as the provided dose. Whey: 20 g Nutri Whey ^TM^ isolate, Canola native: 20 g CanolaPRO^®^ isolate, Canola enzyme: 20 g canola protein isolate processed with peptidyl arginase deiminase enzyme, Canola heat: 20 g canola protein isolate processed at 90 °C

*EAA* essential amino acid, *NEAA* non-essential amino acid, *AA* amino acid

### Gastrointestinal (dis)comfort

Subjects were asked to fill out visual analog scales (VAS) to assess gastrointestinal (GI) comfort. The VAS consisted of 19 questions. Each question started with ‘’to what extent do you experience … right now?’’ and was answered by ticking a 100 mm line (0 mm = not at all, 100 mm = very much). The questions consisted of 8 items related to upper GI discomfort (nausea, general stomach pain, belching, urge to vomit, heartburn, stomach cramps, feeling of fulness, feeling of hunger), 5 items related to lower GI discomfort (flatulence, urge to defecate, intestinal cramps, diarrhea, constipation) and 6 items related to other GI symptoms (dizziness, headache, urge to urinate, bloated feeling, dry mouth, thirst).

### Proteins and processing

Canola protein isolates were supplied by dsm-firmenich AG (Delft, The Netherlands) and whey protein isolate was supplied by FrieslandCampina (Nutri Whey ^TM^ Isolate, FrieslandCampina, Amersfoort, The Netherlands). In short, native canola protein isolate was obtained by cold-pressing rapeseeds to preserve the native state of the proteins in subsequent aqueous extractions. These extractions involved mixing the press cake with salt solution (meal: water ratio of 1:5–1:20) comprising 1–5% NaCl (w/w) at 40–75 °C for 30–60 min, followed by separation of the protein-rich solution from the insoluble material. Next, the pH of the extract was adjusted (range 2–12) followed by clarification using citric acid and/ or ascorbic acid buffers, to remove non-protein substances. Subsequently, a solid/liquid separation step followed by ultrafiltration/diafiltration was used to wash the extract and remove anti-nutritional factors (Mupondwa et al. 2018). Production of the processed canola proteins was performed from the same batch as the native canola protein. Enzymatic treatment of canola protein was based on protein citrullination, which is the post-translational conversion of arginine residues to citrulline, which is linked to better sensory aspects of canola protein. In short, enzyme processed canola protein was prepared by dissolving 5% (w/v) native canola protein in osmosed water at 50 °C. The pH was adjusted to 6.0 and 0.17 U/mL peptidylarginine deiminase enzymewas added, and the solution was incubated for 30 min at 50 °C in an double-jacked tank agitated with three axial impellers at 160 rpm. The enzyme was inactivated for 30 min at 65 °C. The resulting suspension was spray dried (Extractis Spray Dryer, Dury, France) using an inlet temperature of 150 °C and an outlet temperature of 50 °C. Heat processed canola protein was produced by dissolving the native canola protein in osmosed water at 55 °C in a double-jacked tank with three axial impellers at 160 rpm. The product was left to hydrate overnight at 7 °C, mildly stirred at 80 rpm. Thereafter, temperature was increased to 90 °C and maintained for 10 min, then decreased to 60 °C and the mixture was treated in an in-line high shear mixer (ULTRA-TURRAX UTL 2000 Disperser; IKA, Staufen, Germany) to break up protein aggregates. The resulting suspension was spray dried (Extractis Spray Dryer, Dury, France) using an inlet temperature of 150 °C and an outlet temperature of 50 °C.

### Protein analyses

Amino acid contents of the protein powders were analyzed by Eurofins in compliance with requirements in DS EN ISO/IEC 17,025 DANAK 581. In short, acid hydrolysis (ISO 13903:2005; EU 152/2009), oxidation-hydrolysis to measure cysteine and methionine (ISO 13903:2005; EU 152/2009), and alkaline hydrolysis to measure tryptophan (EU 152/2009) were applied in triplicate. The amino acid compositions of all four protein drinks are presented in Table 2. The protein content was determined to be 85.7% for whey protein isolate, 84.3% for native canola protein isolate and 80.9% for both the heat- and enzyme processed canola protein isolates. To provide equivalent doses of 20 g of total protein (based on the sum of total amino acids), the following amounts of powder were dissolved in 300 mL of water: 23.3 g, 23.5 g and 23.5 g for whey protein, native canola protein and both processed canola protein isolates, respectively.

### Plasma analyses

Plasma glucose and insulin concentrations were analyzed using commercially available kits (ref. no. A11A01667, Glucose HK CP, ABX Diagnostics, Montpellier, France; and ref. no. K151BZC-3, Human Insulin Kit, Meso Scale Discovery, Rockville, MD, United States, respectively). Plasma amino acid concentrations were determined by ultra-performance liquid chromatography-mass spectrometry (UPLC-MS; ACQUITY UPLC H-Class with QDa; Waters, Saint-Quentin, France). Specifically, 50 µL blood plasma was deproteinized using 100 µL of 10% SSA with 50 µM of MSK-A2 internal standard (Cambridge Isotope Laboratories, Massachusetts, USA). Subsequently, 50 µL of ultra-pure demineralized water was added and samples were centrifuged (15 min at 21,000 *g*). After centrifugation, 10 µL of supernatant was added to 70 µL of Borate reaction buffer (Waters, Saint-Quentin, France). In addition, 20 µL of AccQ/Tag derivatizing reagent solution (Waters, Saint/Quentin, France) was added after which the solution was heated to 55 °C for 10 min. An aliquot of 1 µL was injected and measured using ultraperformance liquid chromatograph mass spectrometry.

### Statistical analyses

All data are expressed as mean ± SD (standard deviation). Time-dependent variables were analyzed by two-factor repeated-measures ANOVA with both time and treatment as within-subjects factor. Analyses were carried out for the period right before protein ingestion (t = 0 min) until the end of the experimental trial (t = 300 min). In case of significant *Time x Treatment* interaction, individual timepoints were analyzed using a one-way ANOVA with the time points as the dependent variable and treatment as the independent variable. Trapezoidal rule adjusted to baseline concentrations (t = 0 min) was applied to calculate the incremental area under the curve (iAUC) of the amino acid concentrations. Non-time-dependent variables (i.e., iAUC, peak concentrations, time to peak) were compared between treatments using a one-way repeated measures ANOVA. All reported P-values were adjusted using the Bonferroni-Holm method to correct for multiple comparisons (Holm 1979). Statistical significance was set at *P* < 0.05 (Houtvast et al. 2024). All calculations were performed using SPSS (version 29.0, IBM Corporation).

## Results

### Gastrointestinal (dis)comfort

Subjects reported upper GI (nausea, general stomach pain, belching, urge to vomit, heartburn, stomach cramps, feeling of fulness, feeling of hunger) and other GI issues (dizziness, headache, urge to urinate, bloated feeling, dry mouth, thirst) following ingestion of the protein drinks. These symptoms all displayed significant differences over time (Upper: *P* = 0.005, other: *P* < 0.001, respectively). For all assessed GI complaints (upper, lower, other) no differences between treatments (*P = 0.492*, *P* = 0.487, *P* = 0.444) or Time x Treatment interactions (*P = 0.565*, *P* = 0.369, and *P* = 0.514) were reported.

### Plasma glucose and insulin concentrations

**Fig. 1:**
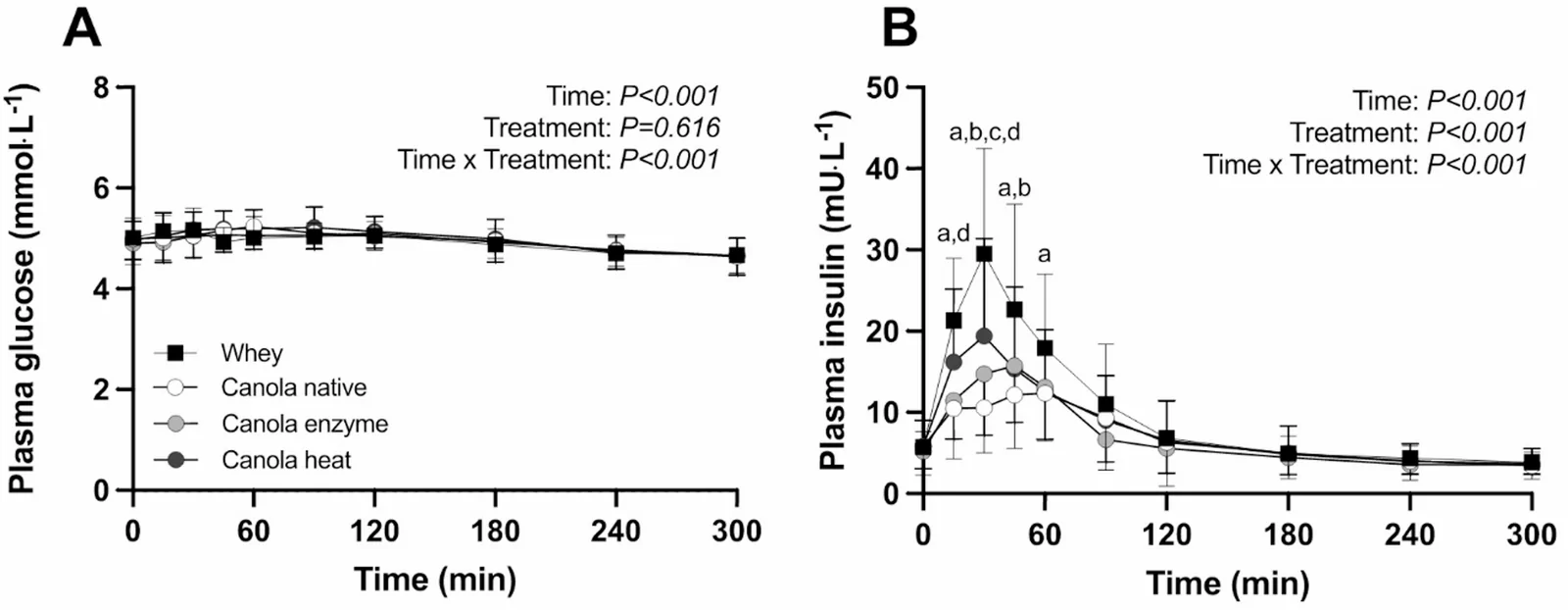
Plasma glucose (**A**) and insulin (**B**) concentrations during the 5 h postprandial period following protein ingestion. Following collection of a baseline blood sample, a protein drink providing 20 g of protein was ingested. Whey: 20 g Nutri Whey ^TM^ isolate, Canola native: 20 g CanolaPRO^®^ isolate, Canola enzyme: 20 g canola protein isolate processed with peptidyl arginase deiminase enzyme, Canola heat: 20 g canola protein isolate processed at 90 °C. Values represent means ± SD

Plasma glucose concentrations were not different between treatments directly prior to protein ingestion (t = 0 min, Fig. 1A; *P* = 0.413). Following protein ingestion, plasma glucose concentrations decreased over time (Time, *P* < 0.001) with no differences between treatments (Treatment, *P* = 0.616). Plasma insulin concentrations were not different between treatments prior to protein ingestion (Fig. 1B; *P* = 0.604). Plasma insulin concentrations were significantly greater following whey compared to native canola protein ingestion (up to t = 45 min, *P* < 0.001). Ingestion of enzyme- and heat processed canola protein resulted in greater plasma insulin concentrations when compared to native canola protein (t = 15 to 45 min, both *P* < 0.001).

### Plasma amino acid concentrations

**Fig. 2:**
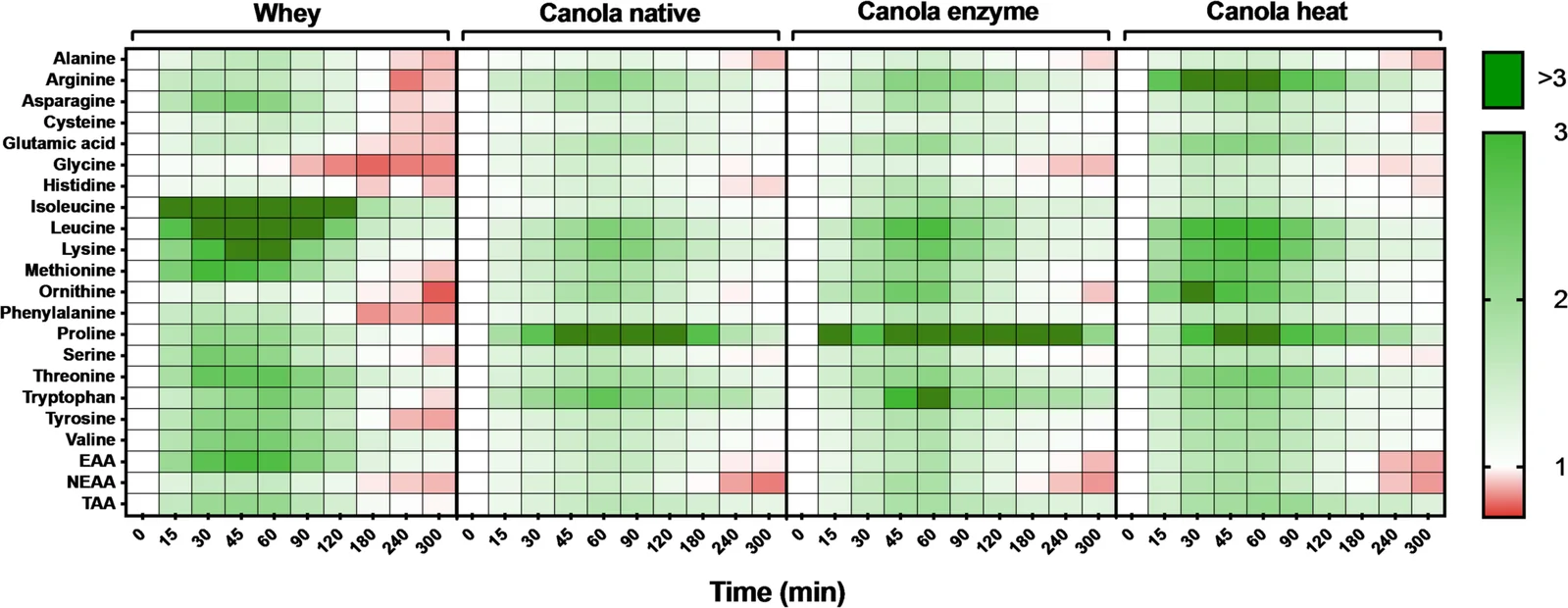
Postprandial plasma amino acid concentrations following ingestion of 20 g native, enzyme-, or heat-processed canola protein isolate, or 20 g whey protein isolate. The fold changes were compared with baseline (t = 0 min, value set to 1) for all timepoints and all conditions. White: no changes in plasma amino acid concentrations when compared with baseline at the indicated time point. Green: plasma amino acid concentrations are elevated above baseline levels. Red: amino acid concentrations are lower compared to baseline. *EAA* essential amino acids, *NEAA* non-essential amino acid, *TAA* total amino acids

Concentrations for all measured amino acids over the 5 h postprandial period are visualized in a heat map displaying the fold changes in plasma amino acid concentrations following protein ingestion when compared to baseline values (t = 0 min values set to 1, Fig. 2).

**Fig. 3:**
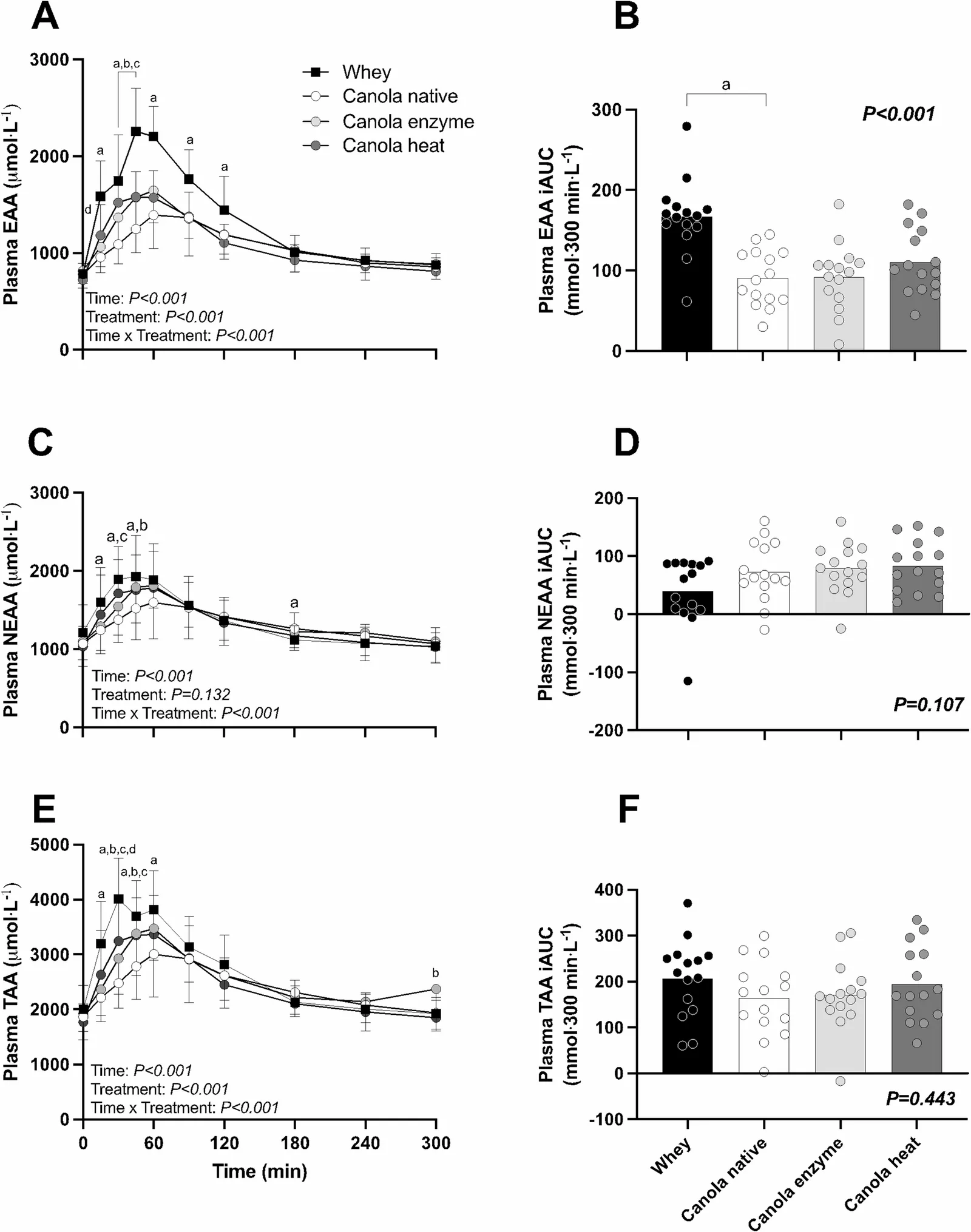
Postprandial plasma EAA (**A**), NEAA (**C**), TAA (**E**) concentrations during the 5-h postprandial period and their incremental area under the curve (iAUC, panel **B**, **D** and **F**) following ingestion of 20 g whey protein isolate, 20 g native canola protein isolate, 20 g enzyme-processed canola protein isolate (Canola enzyme) or 20 g heat-processed canola protein isolate (Canola heat). Values represent means ± SD. Data for plasma amino acid concentrations were analyzed using two-factor repeated-measures analysis of variance. Data for iAUC were analyzed by one-way analysis of variance. Bonferroni-Holm post-hoc testing was applied to locate differences between treatments at each separate time point. ‘’a’’ denotes a significant difference between whey and native canola protein, ‘’b’’ denotes a significant difference between native canola protein and enzyme-processed canola protein, ‘’c’’ denotes a significant difference between native canola protein and heat-processed canola protein, ‘’d’’ denotes a significant difference between the processed canola proteins. Significance was set at *P* < 0.05

Plasma essential amino acid (EAA), non-essential amino acid (NEAA), and total amino acid (TAA) concentrations and their 5 h postprandial amino acid availability (iAUC) are presented in Fig. 3. Plasma EAA concentrations strongly increased following protein ingestion (Fig. 3A, Time; *P* < 0.001), with less of an increase following native canola protein when compared to whey protein ingestion (Time x Treatment, *P* < 0.001). Ingestion of native canola protein resulted in significantly lower peak plasma EAA concentrations compared to whey protein 1492 ± 388 vs. 2367 ± 410 µmol∙L^− 1^, *P* < 0.001) which were also reached later (70 ± 34 vs. 47 ± 23 min, *P < 0.001).* Ingestion of both enzyme- and heat processed canola protein did not result in higher peak plasma EAA concentrations when compared to native canola protein (1687 ± 345, 1674 ± 266, 1492 ± 388 µmol∙L^− 1^, *P* = 0.060 and *P* = 0.116, respectively*).* The time to reach peak plasma EAA concentrations was also not different following the ingestion of enzyme- and heat processed canola protein compared to native canola protein (44 ± 26, 60 ± 14, 70 ± 23 min, *P* = 0.064 and *P* = 0.252, respectively*).* The overall increase in EAAs over the entire 5 h postprandial period, expressed as iAUC, was 46% less following native canola compared to whey protein ingestion (91 ± 35 vs. 167 ± 47 mmol∙300 min∙L^− 1^, *P* < 0.001, Fig. 3B). Enzyme and heat processing of canola protein resulted in 1.5% and 22% greater EAA iAUC when compared to native canola protein (92 ± 42 and 111 ± 41 vs. 91 ± 35 mmol∙300 min∙L^− 1^) but these differences did not reach statistical significance (*P = 0.912* and *P* = 0.282, respectively, Fig. 3B).

Plasma NEAA concentrations increased following protein ingestion (Fig. 3C, Time; *P* < 0.001), with less of an increase following native canola when compared to whey protein ingestion (Time x treatment, *P* < 0.001). Peak plasma NEAA concentrations were not different following native canola versus whey protein ingestion (1712 ± 444 vs. 2084 ± 488 µmol∙L^− 1^ respectively, *P* = 0.068). The time to reach peak plasma NEAA concentrations was not different following native canola versus whey protein ingestion (47 ± 19 vs. 60 ± 36 min, *P* = 0.526). The ingestion of both enzyme- and heat processed canola protein resulted in augmented plasma NEAA concentration increases within the first hour following protein ingestion when compared to native canola protein. Despite this, peak plasma NEAA concentrations following the ingestion of enzyme and heat processed canola protein did not significantly differ from native canola protein (1918 ± 413 and 1909 ± 503 vs. 1212 ± 444 µmol∙L^− 1^, *P* = 0.072 and *P* = 0.288, respectively, Fig. 3C). In line, the time to reach peak plasma NEAA concentrations was not different between enzyme- and heat processed canola protein compared to native canola protein (64 ± 17 and 54 ± 19 vs. 47 ± 19 min, *P* = 0.052 and *P* = 0.290, respectively). The overall increase in NEAAs over the entire 5 h postprandial period, expressed as iAUC (Fig. 3D), was lowest following whey protein ingestion (40 ± 57 mmol∙300 min∙L^− 1^) and increased following the ingestion of native, enzyme- and heat processed canola protein (73 ± 52 vs. 79 ± 43 vs. 84 ± 44 mmol∙300 min∙L^− 1^) but these differences were not statistically significant (Treatment, *P* = 0.107, Fig. 3D).

Overall, TAA concentrations strongly increased following protein ingestion (Time, *P* < 0.001, Fig. 3E), with less of an increase following native canola protein when compared to whey protein ingestion (Time x treatment, *P* < 0.001). The ingestion of native canola protein resulted in significantly lower peak plasma TAA concentrations compared to whey protein (3191 ± 794 vs. 4429 ± 843 µmol∙L^− 1^, *P* < 0.001). The time to reach peak plasma TAA concentrations was not different following native canola compared to whey protein ingestion (44 ± 14 vs. 60 ± 37 min, *P* = 0.157). The ingestion of both enzyme- and heat processed canola protein augmented plasma TAA kinetics resulting in greater circulating levels of TAA within the first hour following protein ingestion compared to native canola. Peak plasma TAA concentrations were greater for enzyme processed canola vs. native canola protein (3599 ± 687 vs. 3191 ± 794 µmol∙L^− 1^, *P* = 0.045). However, peak TAA concentrations following ingestion of heat processed canola protein were not different from native canola protein (3565 ± 722 vs. 3191 ± 794 µmol∙L^− 1^, *P* = 0.166). The overall increase in TAAs over the entire 5 h postprandial period, expressed as iAUC, was lower following native canola compared with whey protein ingestion (163 ± 81 vs. 207 ± 85 mmol∙300 min∙L^− 1^), but this difference was not statistically significant (Treatment, *P* = 0.433). Additionally, ingestion of enzyme- and heat processed canola protein did not result in different TAA iAUC (Fig. 3F) when compared to native canola protein (171 ± 76 and 194 ± 82 vs. 163 ± 81 mmol∙300 min∙L^− 1^ respectively).

**Fig. 4:**
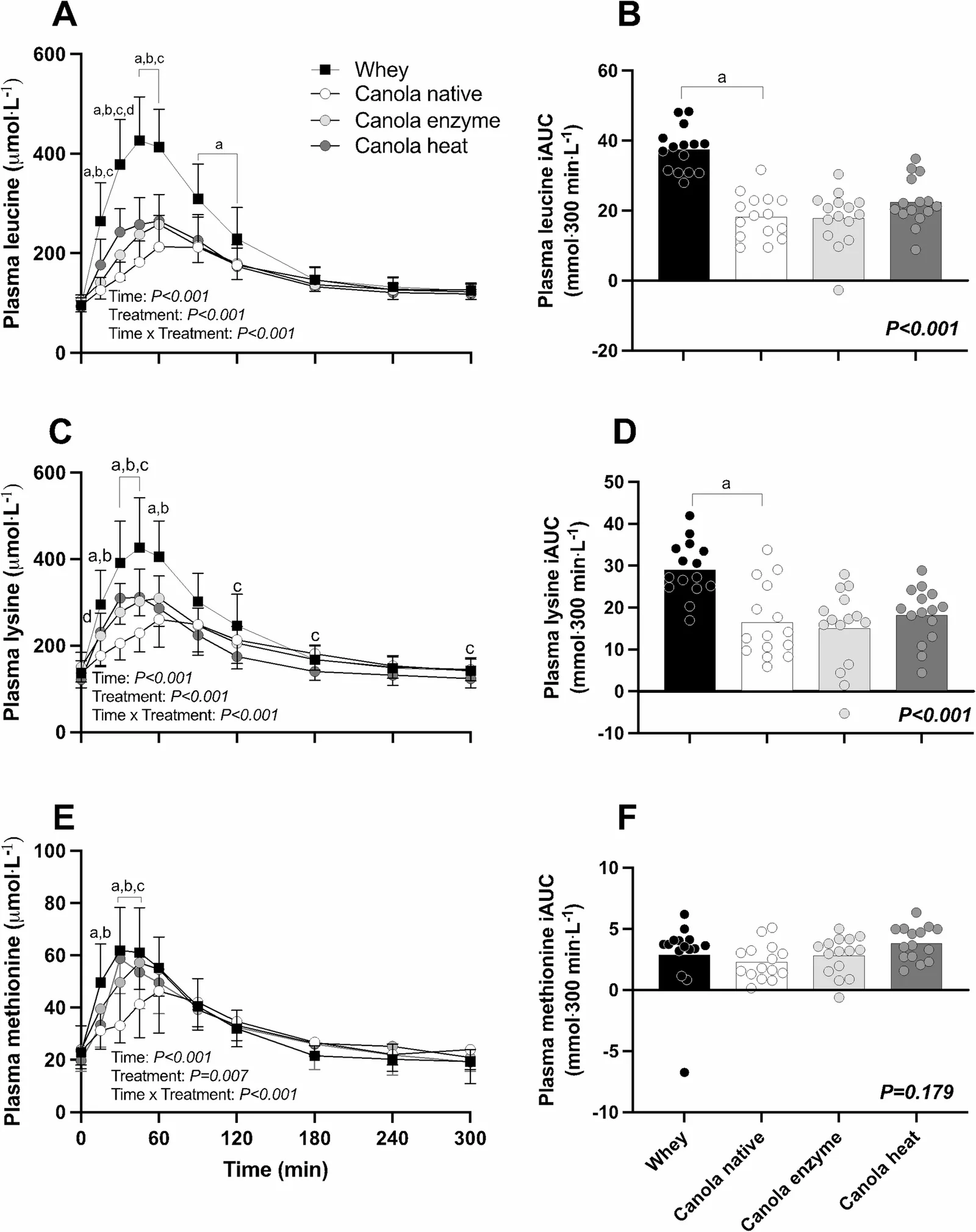
Postprandial plasma leucine (**A**), lysine (**C**), methionine (**E**) concentrations during the 5 h postprandial period and their incremental area under the curve (iAUC, panels **B**, **D** and **F**) following ingestion of 20 g whey protein isolate, 20 g native canola protein isolate, 20 g enzyme-processed canola protein isolate (Canola enzyme), or 20 g heat-processed canola protein isolate (Canola heat). Values represent means ± SD. Data for plasma amino acid concentrations were analyzed using two-factor repeated-measures analysis of variance. Data for iAUC were analyzed by one-way analysis of variance. Bonferroni-Holm post-hoc testing was applied to locate differences between treatments at each separate time point. ‘’a’’ denotes a significant difference between whey and native canola protein, ‘’b’’ denotes a significant difference between native canola protein and enzyme-processed canola protein, ‘’c’’ denotes a significant difference between native canola protein and heat-processed canola protein, ‘’d’’ denotes a significant difference between the processed canola proteins. Significance was set at *P* < 0.05

Plasma leucine concentrations increased over time following protein ingestion (Fig. 4A, Time; *P* < 0.001). Ingestion of native canola protein resulted in lower peak plasma leucine concentrations compared with whey protein (229 ± 67 vs. 448 ± 87 µmol∙L^− 1^ respectively; *P* < 0.001). The time to reach peak leucine concentrations was also longer following native canola compared to whey protein ingestion (71 ± 16 vs. 51 ± 14 min, *P* < 0.001). The ingestion of both enzyme- and heat processed canola protein resulted in greater peak plasma leucine concentrations when compared to native canola protein (262 ± 51 and 279 ± 47 µmol∙L^− 1^ vs. 229 ± 67 µmol∙L^− 1^, *P* = 0.012 and *P* = 0.020 respectively). However, peak plasma leucine concentrations were reached earlier following ingestion of heat processed canola when compared to enzyme processed canola protein (53 ± 20 vs. 57 ± 6 min respectively, *P = 0.004).* Leucine availability over the complete 5 h postprandial period, expressed as iAUC (Fig. 4B), was 51% less following native canola compared to whey protein ingestion (18 ± 6 vs. 37 ± 6 mmol∙300 min∙L^− 1^, *P* < 0.001). Leucine iAUC was not different for enzyme processed canola protein compared to native canola protein (18 ± 8 vs. 18 ± 6 mmol∙300 min∙L^− 1^, *P* = 0.846). Heat processing of canola protein resulted in a 22% greater leucine availability compared to native canola protein, but this difference was not statistically significant (22 ± 7 vs. 18 ± 6 mmol∙300 min∙L^− 1^, *P = 0.177)*.

Plasma lysine concentrations increased over time following protein ingestion (Time; *P < 0.001;* Fig. 4C). Peak plasma lysine concentrations were lower following native canola compared to whey protein ingestion (280 ± 67 vs. 446 ± 110 µmol∙L^− 1^; *P* < 0.001). The time to reach peak lysine concentrations was significantly greater following native canola when compared to whey protein ingestion (78 ± 34 vs. 43 ± 10 min, respectively; *P* = 0.004). Ingestion of both enzyme- and heat processed canola protein tended to result in higher peak lysine concentrations when compared to native canola protein (325 ± 60 and 326 ± 56 vs. 280 ± 67 µmol∙L^− 1^; *P* = 0.054 and *P* = 0.098 respectively). Lysine availability over the complete 5-h postprandial period, expressed as iAUC (Fig. 4D), was 43% less following native canola compared to whey protein ingestion (16 ± 9 vs. 29 ± 7 mmol∙300 min∙L^− 1^, *P* < 0.001). Compared to native canola protein, the ingestion of enzyme-processed canola protein resulted in 8% lower plasma lysine availability, but this difference was not statistically significant (16 ± 9 vs. 15 ± 9, mmol∙300 min∙L^− 1^, *P* = 0.664). In contrast, the ingestion of heat processed canola protein resulted in 11% greater plasma lysine availability compared to native canola, however this difference also did not reach statistical significance (16 ± 9 vs. 18 ± 7 mmol∙300 min∙L^− 1^, *P* = 0.828).

Plasma methionine concentrations increased over time following protein ingestion (Time; *P* < 0.001, Fig. 4E). Peak plasma methionine concentrations were lower following native canola when compared to whey protein ingestion (52 ± 15 vs. 68 ± 17 µmol∙L^− 1^; *P* = 0.048). The time to reach peak methionine concentrations was significantly greater following native canola when compared to whey protein ingestion (70 ± 24 vs. 41 ± 17 min, respectively; *P* = 0.032). Ingestion of enzyme- and heat processed canola protein did not result in different peak methionine concentrations when compared to native canola protein (62 ± 13 and 63 ± 15 vs. 52 ± 15 µmol∙L^− 1^, *P* = 0.123 and *P* = 0.154, respectively*)*. Methionine availability over the complete 5 h postprandial period, expressed as iAUC (Fig. 4F), was 20% less following native canola protein when compared to whey protein ingestion (2 ± 1 vs. 3 ± 3 mmol∙300 min∙L^− 1^). The ingestion of enzyme- and heat processed canola protein resulted in 22% and 66% greater plasma methionine availability compared to native canola protein (3 ± 2 and 4 ± 1 vs. 2 ± 1 mmol∙300 min∙L^− 1^, respectively) but none of these differences reached statistical significance (Treatment, *P* = 0.150).

In general, increases in plasma concentrations of other measured amino acids revealed significant differences over time following ingestion of whey and all three canola proteins (Time x Treatment, all *P* < 0.001) for all measured amino acids. The increases in plasma amino acid concentrations over the 5 h postprandial period (iAUC) were different following whey vs. native canola protein in case of arginine, glycine, isoleucine, ornithine, threonine, tryptophan, tyrosine and valine (all *P* < 0.001). For all other measured amino acids, the iAUC did not differ between treatments (Supplemental Fig. 1).

## Discussion

The present study compared postprandial plasma amino acid profiles following the ingestion of native, heat-processed, and enzyme-processed canola protein as well as whey protein in healthy, young females. The ingestion of whey protein resulted in a more rapid and pronounced increase in circulating amino acids when compared to native canola protein. Ingestion of both enzyme- and heat processed canola protein accelerated the postprandial increase in circulating amino acids, but did not result in greater overall plasma amino acid availability during a 5 h postprandial period when compared to the ingestion of native canola protein.

Plant-derived proteins are generally considered of low(er) quality when compared to animal-derived proteins due to differences in protein digestion and amino acid absorption kinetics and a less balanced amino acid profile (Gorissen et al. 2018; Vliet et al. 2015; Pinckaers et al. 2021). In contrast to many plant-derived proteins (Gorissen et al. 2018; Pinckaers et al. 2021; Bailey et al. 2023), canola-derived protein isolate has a well-balanced amino acid profile with no apparent deficiencies in lysine and methionine. In Table 2, we compared the amino acid composition of native canola protein with whey as a high-quality, animal-derived, reference protein. Canola protein isolate contained a higher proportion of NEAA (63% vs. 52%) but lower proportion of EAA (37% vs. 48%) compared with whey protein. These compositional differences were reflected in the postprandial plasma amino acid responses (Fig. 3) and likely contributed to the lower amino acid availability observed with native canola protein. The ingestion of native canola protein resulted in an attenuated rise in circulating plasma essential amino acids. This was accompanied by a delay in the response, with peak leucine concentrations being reached 20 min later following ingestion of canola compared with whey protein (51 ± 14 vs. 71 ± 16 min, *P* < 0.001, Fig. 4A). In line with lower and more delayed postprandial increases in plasma essential amino acids, the overall postprandial plasma essential amino acid availability was also significantly less following ingestion of native canola when compared to whey protein (91 ± 35 vs. 167 ± 47 mmol∙300 min∙L^− 1^, *P* < 0.001, Fig. 3B). The attenuated postprandial rise in circulating amino acids following ingestion of plant- versus animal-derived proteins has been reported previously (Gorissen et al. 2016; Tang et al. 2009; Yang et al. 2012; Pinckaers et al. 2022, 2024a, b) and may be attributed to differences in protein structure and function that stimulate amino acid retention in splanchnic tissues (Groen et al. 2015; Trommelen et al. 2021; Kashyap et al. 2018, 2019). Lower postprandial protein-derived amino acid availability lowers protein bioavailability and may even compromise the bio-functionality of a protein or protein source (Trommelen et al. 2023). Therefore, many strategies are being applied to accelerate protein digestion and subsequent amino acid absorption.

Protein processing can be used to modulate protein digestion and amino acid absorption, thereby increasing postprandial plasma amino acid availability (Calbet and Holst 2004). Previously, enzymatic hydrolysis of casein has been shown to accelerate protein digestion and increase protein-derived amino acid release in the circulation, resulting in greater overall postprandial plasma amino acid availability in vivo in humans In the present study, protein processing, either through enzymatic modification or heating, was applied to the native canola protein to study the effects on protein digestion and amino acid absorption. Here, we observed a more rapid postprandial rise in the concentration of some, but certainly not all, amino acids following ingestion of enzyme processed versus native canola, but this did not result in greater overall postprandial plasma amino acid availability (171 ± 76 vs. 163 ± 81 mmol∙300 min∙L^− 1^ respectively, Treatment, *P* = 0.433; Fig. 3F). So far, only one study has compared postprandial plasma amino acid responses following ingestion of a hydrolyzed versus intact canola protein (Fleddermann et al. 2013). Fleddermann et al. reported more rapid increases in circulating amino acid concentrations following ingestion of hydrolyzed versus intact canola protein, without differences in overall plasma amino acid availability. Besides enzymatic hydrolysis, heating can also be applied to proteins with relative low denaturation temperatures. The open protein structure obtained through heating can enhance digestibility and accelerate amino acid absorption kinetics (Evenepoel et al. 1998; Fuchs et al. 2022; Bax et al. 2012, 2013; Mansour et al. 1993; Badshah et al. 1993). Previous work in pigs has shown that heat processing can improve canola protein digestibility by 22% (Bailey et al. 2023). In the present study, ingestion of heat processed canola protein resulted in a more rapid postprandial rise in some of the circulating amino acid concentrations. Even though we also observed a 22% greater increase in plasma essential amino acid availability following the ingestion of heat processed versus intact canola protein (111 ± 41 vs. 91 ± 35 mmol∙300 min∙L^− 1^, respectively, Fig. 3B), no significant differences were observed between treatments (*P = 0.282*). Overall, the present data show a modest impact of canola protein processing on the postprandial increase in circulating plasma amino acid concentrations, without any substantial changes in the overall postprandial increase in plasma amino acid availability.

Despite the absence of any substantial impact of the currently tested enzyme or heat processing conditions of canola protein isolate on the postprandial rise in circulating plasma amino acid concentrations, it remains important to evaluate the effects of various processing techniques on protein digestion and subsequent amino acid absorption. Here we assessed the postprandial rise in circulating amino acids, expressed as the incremental area under the curve (iAUC), as a proxy for postprandial protein-derived amino acid availability. However, a more quantitative assessment of protein digestion and amino acid absorption requires a dual isotope tracer approach (Trommelen and Loon 2024). The use of intrinsically labeled proteins would be required to provide evidence for the proposed impact of protein processing on protein digestion and amino acid absorption in vivo in humans (Groen et al. 2015; Lieshout et al. 2025; Koopman et al. 2009; Loon et al. 2009). Here, we show no significant differences in postprandial plasma amino acid availability following ingestion of both enzyme and heat processed canola protein when compared to the ingestion of native canola protein. However, it should be noted that protein processing techniques may affect specific amino acids. For example, previous work in our group has shown that heating of milk protein can cause glycation, which strongly reduces protein-derived lysine (bio)availability (Nyakayiru et al. 2020; Lieshout et al. 2025). Therefore, we also evaluated individual plasma amino acid concentrations and their incremental areas under the curve (Supplemental File 1). In contrast to milk protein, heat processing of canola protein did not negatively impact postprandial plasma lysine availability, and no specific differences in individual amino acid responses were observed following the ingestion of processed versus native canola protein.

The present data show a minor role of enzymatic modification and mild heat treatment on postprandial plasma amino acid bioavailability following canola protein ingestion. However, as commercial protein processing techniques vary considerably, the impact of commonly applied processing techniques on postprandial amino acid bioavailability and bio-functionality will need to be addressed (Juul et al. 2022). This may be of increasing relevance with regards to the protein transition towards the consumption of more plant-based proteins (Willett et al. 2019). The use of plant-derived protein isolates and concentrates in meat and dairy alternatives requires extensive processing and may strongly impact the bioavailability of the available protein(s) in these products. Therefore, more work is needed to assess the impact of the various protein- and food processing procedures on the bioavailability of these proteins or protein sources in vivo in humans.

In conclusion, ingestion of native canola protein allows for a more delayed postprandial rise in circulating essential and non-essential amino acids and a lower postprandial plasma amino acid availability when compared to the ingestion of whey protein. Ingestion of enzyme-modified or heat-processed canola protein accelerates the postprandial rise in circulating amino acids but does not further augment overall plasma amino acid availability throughout a 5 h postprandial period when compared to the ingestion of native canola protein.

## Supplementary Information

Below is the link to the electronic supplementary material.

Supplementary Material 1.

## Data Availability

No datasets were generated or analysed during the current study.
