# Supplementary material for: Evaluation of processing of canola protein isolate on postprandial plasma amino acid profiles in healthy, young females
Source: Amino Acids. 2025 Oct 17;57:50. doi: 10.1007/s00726-025-03482-1 (PMC12534268; doi:10.1007/s00726-025-03482-1)
Supplement: Supplementary file 1 [file 726_2025_3482_MOESM1_ESM.docx]

**SUPPLEMENTAL FIGURES**

**Supplemental Figure 1.** Postprandial plasma amino acid concentrations during the 300 min postprandial period following the ingestion of 20 g whey protein isolate, 20 g native canola protein isolate, 20 g enzyme-processed canola protein isolate (Canola enzyme) or 20 g heat-processed canola protein isolate (Canola heat). Panels B, D, F, H, J, L, N, P, R, T, V, X, Z, AB, AD, AF represent the 0 – 5 h incremental area under curve (iAUC) following ingestion of the proteins. Values represent means ± SD. Data for plasma amino acid concentrations were analyzed using two-factor repeated-measures analysis of variance. Data for iAUC were analyzed by one-way analysis of variance. Bonferroni-Holm post-hoc testing was applied to locate differences between treatments at each separate time point. ‘’a’’ denotes a significant difference between whey and native canola protein, ‘’b’’ denotes a significant difference between native canola protein and enzyme-processed canola protein, ‘’c’’ denotes a significant difference between native canola protein and heat-processed canola protein, ‘’d’’ denotes a significant difference between the processed canola proteins. Significance was set at *P<0.05*.
